# The Insulin Receptor Is Required for the Development of the *Drosophila* Peripheral Nervous System

**DOI:** 10.1371/journal.pone.0071857

**Published:** 2013-09-12

**Authors:** Annie Dutriaux, Aurélie Godart, Anna Brachet, Joël Silber

**Affiliations:** University Paris Diderot, Sorbonne Paris Cité, IJM, UMR 7592 CNRS, Paris, France; University of Dayton, United States of America

## Abstract

The Insulin Receptor (InR) in *Drosophila* presents features conserved in its mammalian counterparts. InR is required for growth; it is expressed in the central and embryonic nervous system and modulates the time of differentiation of the eye photoreceptor without altering cell fate. We show that the InR is required for the formation of the peripheral nervous system during larval development and more particularly for the formation of sensory organ precursors (SOPs) on the fly notum and scutellum. SOPs arise in the proneural cluster that expresses high levels of the proneural proteins Achaete (Ac) and Scute (Sc). The other cells will become epidermis due to lateral inhibition induced by the Notch (N) receptor signal that prevents its neighbors from adopting a neural fate. In addition, misexpression of the InR or of other components of the pathway (PTEN, Akt, FOXO) induces the development of an abnormal number of macrochaetes that are *Drosophila* mechanoreceptors. Our data suggest that InR regulates the neural genes *ac*, *sc* and *sens*. The FOXO transcription factor which is localized in the cytoplasm upon insulin uptake, displays strong genetic interaction with the InR and is involved in Ac regulation. The genetic interactions between the epidermal growth factor receptor (EGFR), Ras and InR/FOXO suggest that these proteins cooperate to induce neural gene expression. Moreover, InR/FOXO is probably involved in the lateral inhibition process, since genetic interactions with N are highly significant. These results show that the InR can alter cell fate, independently of its function in cell growth and proliferation.

## Introduction

Each organ grows by controlling cell number and cell size to reach its final dimensions. This process is tightly regulated and modulated by environmental factors such as nutrient availability.

The *Drosophila* insulin pathway is highly conserved from mammals to *Drosophila*. In *Drosophila* the InR regulates a number of functions linked to metabolism, organ growth, cell number and cell size, and mutants of the pathway present size defects [1], [2]. InR is also essential for normal development and is required for the formation of the epidermis and the central and peripheral nervous systems (PNS) during embryogenesis [3]. It is also expressed in imaginal discs and necessary for cell number specification and cell growth [4].

**Figure 1:**
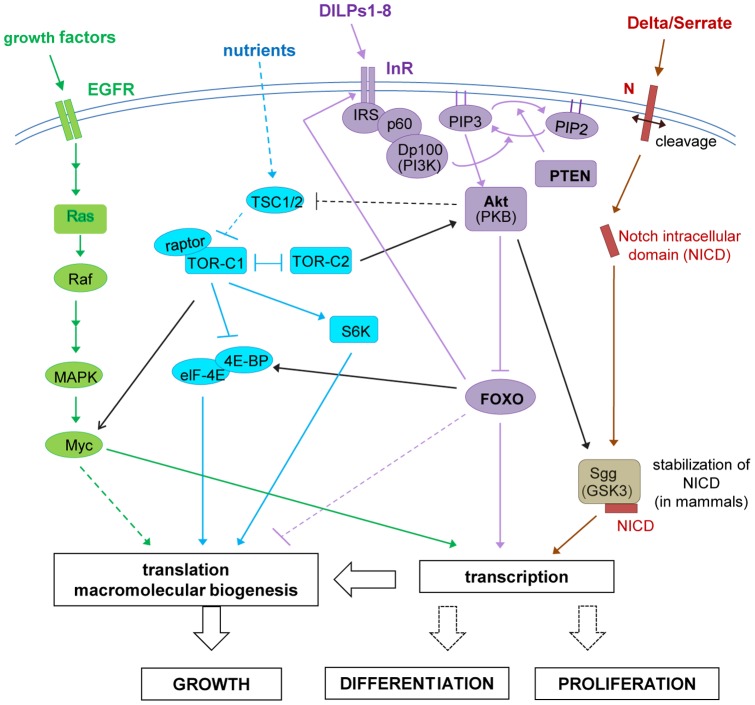
Schematic outline of *Drosophila* InR/TOR signaling. Functional relationships between the InR (purple), TOR (blue), EGFR (green) and N (brown) pathways are indicated with black links. Arrows indicate activation, whereas bar-ended lines indicate inhibitory interactions. Broken lines indicate indirect interactions or interactions requiring further study.

The InR pathway described extensively in Figure 1 is composed of Chico the homologue of the insulin receptor substrates (IRSs), PTEN, which is a phosphatase and therefore an antagonist of the pathway, and the Akt kinase responsible for the phosphorylation of different components of the pathway. The corresponding genes are important for cell size and cell number, except for Akt that affects cell size only [5].

Extracellular ligand homologues to insulin regulate InR activity during development. Eight genes have been identified, *dilp1–8*, for Drosophila insulin-like peptides (DILP). *dilp2* is the most closely related to mature insulin and is the only DILP with broad expression in imaginal discs. Overexpression of *dilp2* increases both cell number and cell size of different organs [2]. A new component, Dilp8, coordinates growth and plasticity of the organs [6] [7].

A major component of the insulin signaling cascade, the transcription factor FOXO is phosphorylated by Akt leading to its cytoplasmic localization and thus inhibition of its transcriptional activity. The *FOXO* mutant lacking Akt phosphorylation sites, remains in the nucleus, and is constitutively active. This is the case of the human *hFOXO^3a-TM^* mutant, which induces growth arrest in *Drosophila* when overexpressed, by specifying cell number but not cell size [8]. FOXO directly regulates the translational regulator 4E-BP (eukaryotic initiation factor 4E binding protein) and InR itself (Figure 1), thus providing a feedback mechanism [9]. This places FOXO as a key transcriptional regulator of the insulin pathway regulating growth [10]. Moreover, microarray experiments in S2 cells identified more than 200 genes in addition to *4E-BP* and *InR* that are regulated by FOXO, confirming that FOXO is a transcriptional regulator in different developmental processes [11], [12], [13].

It has been shown that Akt promotes protein synthesis through TOR-mediated phosphorylation and through inactivation of the translational inhibitor 4E-BP which interacts strongly with the initiation factor eIF-4E. Another component, the tumor suppressor TSC2 is a phosphorylation target of Akt. TOR also phosphorylates the S6 kinase (S6K). *S6K* mutants display developmental delay and reduction in body size with smaller cells. Thus the InR/TOR pathway is finely tuned to be particularly sensitive to nutrients and environmental changes. This is achieved by different intracellular feedback loops ([5] and Figure 1). Another component Rheb regulates Notch and plays a late role in PNS development [14] Neuronal differentiation is under the control of genes that induce proliferation of progenitor cells and then of other genes necessary for the differentiation of these cells. Some genes can achieve both processes. This is the case of the IR (insulin receptor) and the IGF-IR (insulin like growth factor receptor) in vertebrates [15], [16].

It has been shown that the InR/TOR pathway plays a role in controlling timing of neural differentiation, and that activation of this pathway leads to the precocious acquisition of neuronal cell fate, whereas loss of function delays differentiation but does not alter cell fate. This was observed in photoreceptor formation but also in the chordotonal organs of the leg of *Drosophila,* indicating that InR is required for temporal control of development [17].

*InR* null homozygote embryos are defective in the central and PNS [3], but little is known concerning the role of InR in PNS development in larvae. Abnormal adult PNS development can be visualized looking at bristles, microchaetes and macrochaetes that are mechanoreceptors. All bristles have a very stereotype pattern in the adult and are composed of four cells. In particular the 11 pairs of macrochaetes display a constant position and were given individual names. The bristle comprises the shaft, the socket, the neuron and the sheath. These cells are generated by successive divisions from a single sensory organ precursor (SOP) cell via a fixed lineage [18]. The first step in SOP determination is the formation of the proneural cluster (between 20 and 30 cells for macrochaete) that segregates from the ectoderm in the wing imaginal disc. These cells express the proneural genes *ac* and *sc* and form the proneural field. These genes play a key role in the process and allow the cluster to become competent to become a SOP. Inactivation of the genes induces disappearance of some of the macrochaetes while ectopic expression leads to additional bristles. *ac* and *sc* are at the top of the hierarchy of genes involved in macrochaete formation. The SOP is selected from a few cells that accumulate higher concentrations of the proneural proteins (Ac/Sc) than their neighbors and occupy stereotyped positions within the proneural cluster. Cell-cell signaling within the cluster is mediated by N in one cell and Delta (Dl), the ligand, in the other cell [19]. Dl is induced by the Ac/Sc complex and N, through Suppressor of Hairless (Su(H)), activates the Enhancer of Split (E(spl)) complex that then interferes with the proneural genes to activate targets [20], [21]. This is achieved through protein-protein interaction between the E(spl) gene products and the Ac/Sc proteins or by direct binding of E(Spl) to common target genes [22]. In a proneural cluster only cells that present the highest level of proneural proteins can escape inhibition by N signaling. The cells that escape the signal become a SOP and by lateral inhibition prevent the neighboring cells from acquiring the same fate [19], [23]. Proneural gene expression is under the control of enhancers that drive expression of both Ac and Sc in a proneural cluster. For instance the dorsocentral (DC) prepattern, is different from the scutellar (SC) prepattern. These specific enhancers mediating Sc self-stimulation play a central role in SOP determination.

The EGFR signaling pathway plays an important role in macrochaete formation and is activated at different stage of the sensor organ development. The EGFR and Ras which transduces the pathway (Figure 1), positively interact in the proneural cluster that determines SOP emergence, restraining the N signal during macrochaete formation [24].

Here we have investigated the function of the InR/FOXO pathway in bristle development and show that InR and FOXO are necessary for SOP determination, independently of the InR function in growth. A combined role of the EGFR/Ras pathway with the InR pathway is postulated. However the TOR pathway does not seem to be involved in the process suggesting that InR/FOXO acts on other target genes than on *4E-BP*.

The results obtained examining the expression of *ac*, *senseless* (*sens*) and several enhancers of *sc* show that the InR increases the number of SOP in the proneural field independently of its role in cell proliferation, by regulating *ac* and *sc* expression. This is confirmed by genetic interactions between the InR/FOXO and both *sc* and N. The results indicate that the EGFR/Ras pathway could cooperate with InR/FOXO in this process.

## Results

### Requirement for InR for PNS formation in the adult fly thorax

**Figure 2:**
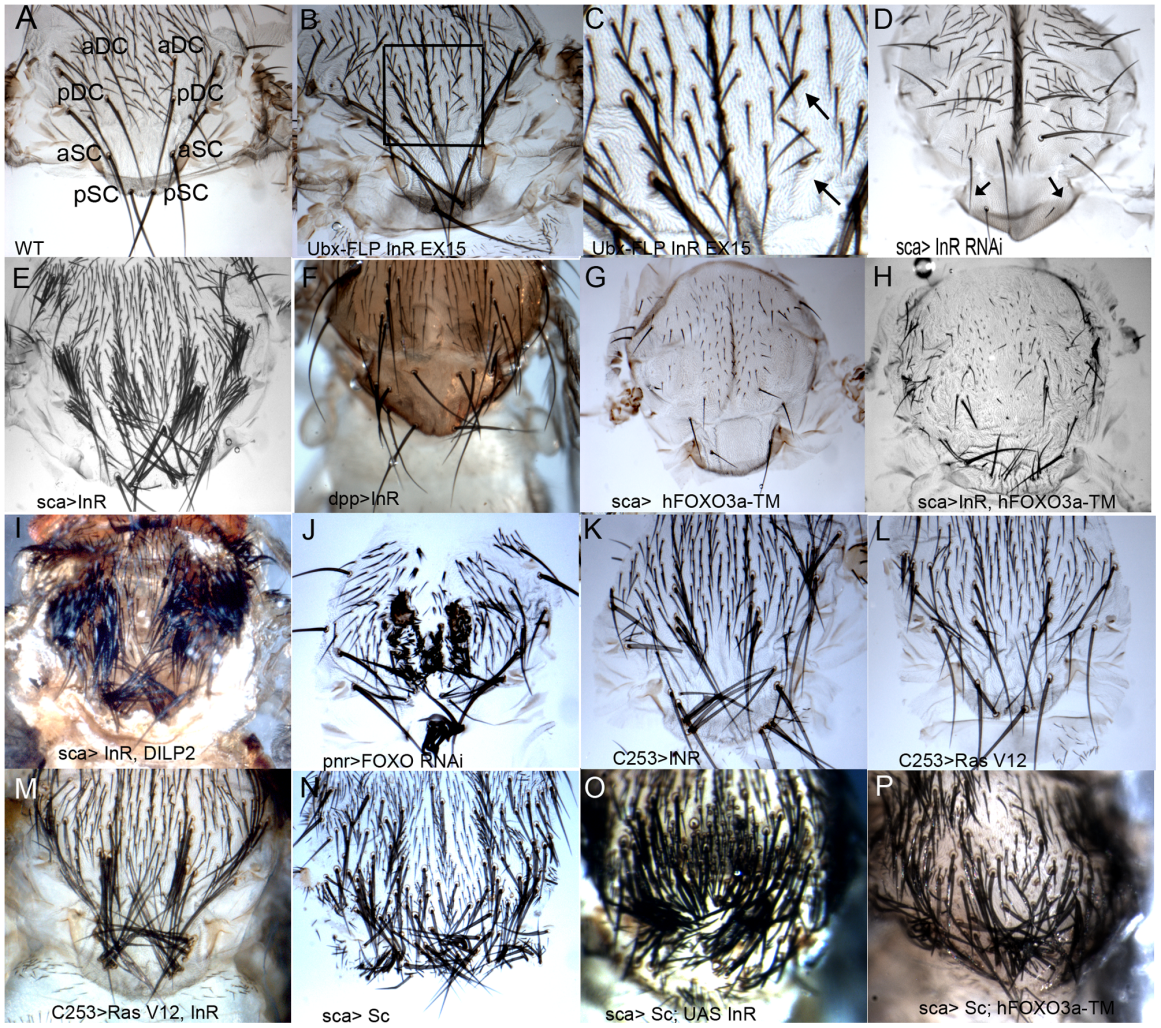
Effects of InR on bristle formation. **A**) Wild-type fly; anterior and posterior DC and SC macrochaetes, designated aDC, pDC and aSC, pSC are shown. **B and C**) *InR* null clones were generated crossing *y,w,Ubx-FLP;FRT82B* and *FRT82B, dInR^EX15^*. Square area magnified showing lack of DC macrochaetes (arrow). In **D** *sca>InR RNAi* notum. Some macrochaetes are missing from the scutellum. Overexpression of *Inr* with *sca-GAL4* (**E)**, *dpp-GAL4* **(F)** or *C253-GAL4* (**K**), led to extra macrochaetes and microchaetes. Note that most of the supplementary macrochaetes have a socket and shaft. In **I** overexpression of both *dilp2* and *InR* in *sca* strongly enhanced the *InR* overexpression phenotype. (**G** and **H**) *FOXO* and *InR* play opposite roles. In **G** lack of macrochaetes is observed on *sca>hFOXO^3a-TM^* flies, and in **H**, it is a decrease of the *InR* overexpression phenotype (compared to **E)** in sca>*Inr*,*hFOXO^3a-TM^*. In **J** *pnr>FOXO RNAi* fly, an increase in microchaetes and macrochaetes on the notum and scutellum are observed. Overexpression of *InR* (**K**), or *Ras^V12^* (**L**), induces extra bristles, effect, stronger with both transgenes (**M**), supporting the hypothesis of genetic interaction between the two pathways. *sca>sc* generates extramacrochaetes **(N)**, a phenotype enhanced by overexpressing *InR* **(O)**, and is decreased by *UAS-hFOXO^3a-TM^* **(P)**.

**Figure 3:**
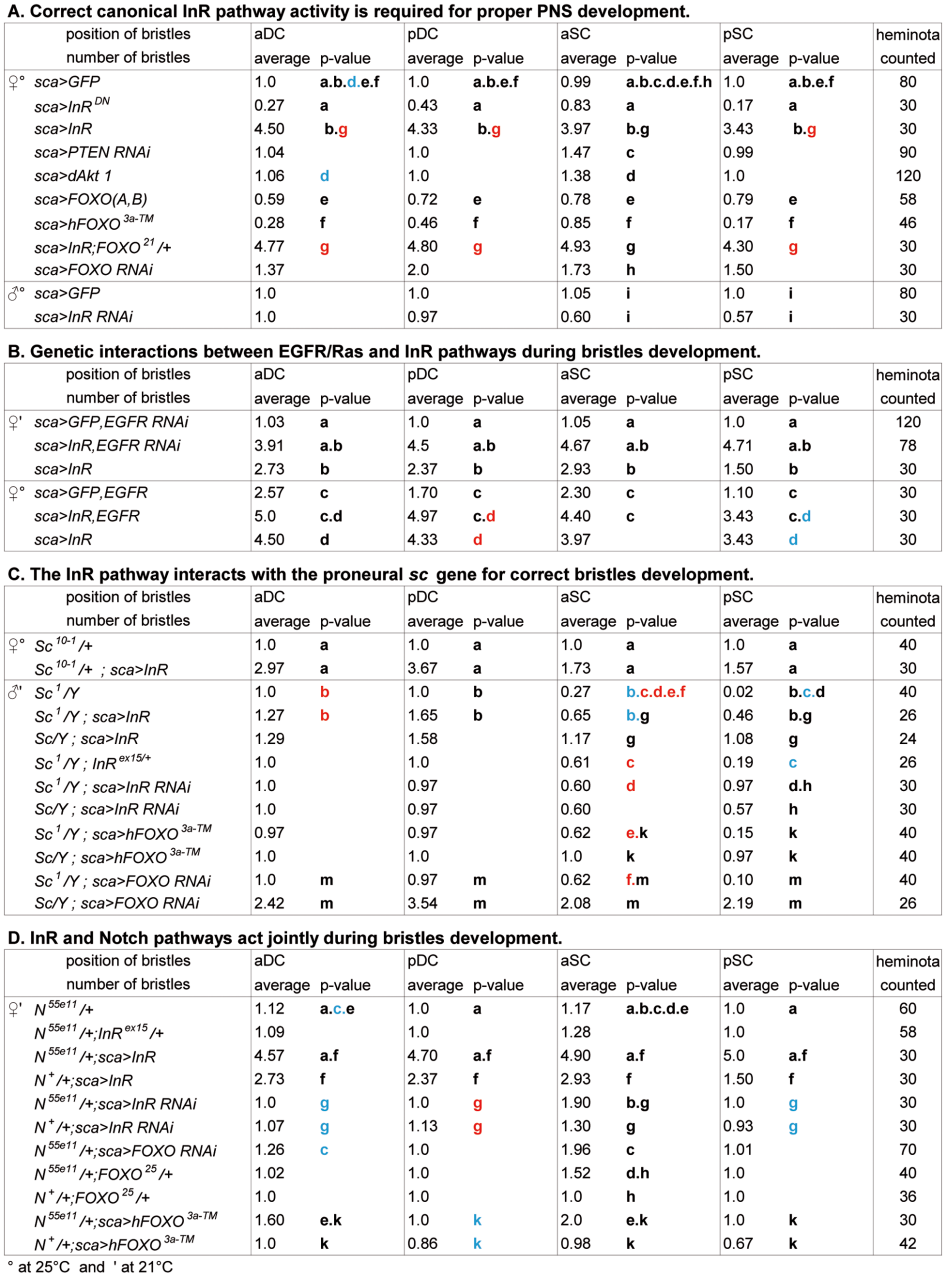
Genetic interactions of *InR* on macrochaete formation. The number of bristles (n) counted on female heminota (except in Table C on male heminota), at aDC, pDC, aSC and pSC positions. The number of macrochaetes for each position was determined and 6 classes were established: n<1, n = 1, n = 2, n = 3, n = 4 or n≥ 5. Experiments were performed at 25°C, except when otherwise stated. A minimum of 24 hemi-thoraces were counted and the results are expressed in the medium percentage of the number of macrochaetes for a given position. The Fisher Exact Test with the “R” programming language was used to calculate p-values on the 6 classes to compare the phenotypes between two classes. Only significant results are indicated, using the following code: blue letters for 0.05<p<0.01, red letters for 0.01<p<0.001 and black letters for p<0.001.

Since *InR* null mutants are mostly lethal at the homozygote state and the few escapers do not show a phenotype at the level of the PNS, we tried to generate null clones, but the clones recovered were usually very small or absent [17]. To recover larger clones the FLP/FRT system was used [25], with Ultrabithorax (Ubx) flipase (*y,w Ubx-Flp; FRT82B*), *FRT82B*, *dInR^EX15^*, which is an *InR* null allele. Flies lacking macrochaetes mainly DC macrochaetes were obtained (Figures 2A–C). To monitor the lack of *InR* function in specific regions of the wing imaginal disc, an *UAS-InR RNAi* construct and an *UAS-InR^DN^* transgene were overexpressed. We focused on the anterior and posterior dorsocentral and scutellar macrochaetes, designated (a) DC and (a) SC. Overexpression of *InR RNAi*, driven by *sca-GAL4* (*scabrous* and designated here *sca>InR RNAi*), in the proneuronal cluster led mainly to lack of SC macrochaetes (Figures 2D and 3A and supporting information S1A). The *InR^DN^* strain driven by *sca-GAL4* resulted in the absence of both (a) DC and (a) SC macrochaetes (Figure 3A).

To assess more precisely the role of InR, overexpression experiments were undertaken. The InR pathway was deregulated in proneuronal clusters with *sca-GAL4*. Different constructs were used: the *UAS-InR^WT^* [26], the *UAS-InR^exel^* (exelexis), and an activated form *UAS-InR^act^* [27]. The overexpression of the three strains, led to supplementary microchaetes and/or macrochaetes. The *UAS-InR^WT^* construct (later called *UAS-InR*) led to the most spectacular phenotype (Figures 2E and 3A), and was used in all following experiments. In general additional macrochaetes were composed of a normal shaft and socket cells but two macrochaetes in the same socket were also observed as well as longer and larger microchaetes. When another driver *decapentaplegic (dpp)-GAL4* was used, ectopic SC macrochaetes could be detected (Figure 2F). These results indicate that the InR acts on upstream genes necessary for macrochaete and microchaete formation. Moreover overexpression of both *dilp2* and *InR* strongly increased the InR overexpression phenotype (Figure 2I), while overexpressing *sca>dilp2* by itself had no effect. The fact that overexpression of the receptor and its ligand enhance the effect of *InR* only is strongly in favor that the phenotype is not due to off-targets effects.

The InR pathway has been studied in detail and therefore we investigated whether other components of the pathway are also involved in PNS formation. PTEN encodes a phosphatase, antagonist of the pathway [28]. Overexpressing *sca>PTEN RNAi* led to an increase of macrochaetes (Figure 3A), as would be expected if PTEN plays a role opposite to that of InR. Additional aSC macrochaetes were observed at 25°C, and at 29°C all the macrochaetes were affected (data not shown).

When Akt a central kinase of the pathway involved in nuclear translocation of FOXO [10] was overexpressed (*sca>Akt*), additional aSC macrochaetes appeared at 25°C (Figure 3A) and more macrochaetes were affected at 29°C (data not shown). These results are in line with *InR* overexpression experiments and support a role of different components of the InR pathway in PNS development.

Upon insulin uptake, FOXO is phosphorylated by Akt leading to its cytoplasmic localization and hence inhibition of its transcriptional activity [10]. Thus it could be expected that InR deregulation and FOXO deregulation have opposite effects on PNS development. Indeed, overexpression of *sca>dFOXOA/B* led to a highly significant lack of both DC and SC macrochaetes, in agreement with an antagonist role of InR and FOXO (Figure 3A). A stronger phenotype was observed with *hFOXO^3a-TM^*, the mutant constitutively located in the nucleus (Figures 2G and 3A). These experiments suggest that InR and FOXO act mostly antagonistically in this process and mainly at the same step of macrochaete development. Moreover the concomitant overexpression of *InR* and *hFOXO^3a-TM^* (noted here *sca>Inr, hFOXO^3a-TM^*) significantly suppresses the InR phenotype (Figure 2H). In addition, when *sca>InR* in a heterozygote *FOXO^21^/+* null mutant background, a highly significant (p<0.01) increase in the number of macrochaetes is observed compared to the overexpression of *InR* only (Figure 3A). This confirms the hypothesis that both *InR* and *FOXO* play a role in macrochaete development.

It has been reported that *FOXO null* homozygote mutants are fully viable and do not display any phenotype and in particular any bristle phenotype [10]. However, in the cross *FOXO^21^/FOXO^25^* some flies displayed additional macrochaetes (supporting information S2) and even *FOXO^21^* heterozygotes (supporting information S1A).

Moreover *FOXO* null clones in which *InR* was overexpressed display a stronger InR phenotype compared to InR overexpressing clones. In addition eye phenotypes with tufted bristles were observed, that do not exist in InR overexpressing clones (supporting information S2). Using an *UAS-FOXO RNAi* strain, *FOXO RNAi* was overexpressed in *sca* and *pannier* (*pnr*) regulatory sequences. Excess aSC macrochaetes were observed with *sca>UAS-FOXO RNAi* (Figure 3A), while with *pnr>UAS-FOXO RNAi* mainly extra microchaetes were detected (Figure 2J). The double heterozygote *InR^EX15^/ FOXO^21^* is completely wild type (data not shown). However, we constructed a double homozygote strain null both for *InR* and *FOXO* that displays at times an excess but generally a lack of macrochaetes (supporting information S1A). This agrees with the hypothesis that most InR phenotypes are due to FOXO cytoplasmic localization. Therefore these experiments agree with a role of FOXO in PNS development and show that the phenotypes observed with InR are opposed to those obtained with nuclear FOXO.

InR is closely linked to the TOR pathway and most of the components of the pathway are required for cell growth ([5] for review). However the complexity of the pathway is increased by feedback loops and interconnections: TOR regulates growth controlling the S6K and eIF-4E activities; in turn eIF-4E interacts with 4E-BP (Figure 1).

Different components of the pathway were tested by under and overexpression experiments. No bristle phenotype appeared with *TSCI*, *S6K*, *Rheb RNAi, raptor RNAi* and *4E-BP* indicating that the phenotypes observed with *InR* are not directly connected with growth (supporting information S1E, S5 and data not shown).

*InR* induces cell growth and proliferation. An important issue is to understand whether the phenotypes observed when *InR* is overexpressed are due to these processes.

Major genes involved in proliferation (*dE2F1*, *dacapo* the *p21* homolog) or in apoptosis (*dIAP* the inhibitor of apoptosis, *p53*) were tested by overexpression experiments alone or also with overexpression of *InR*. No significant interactions were observed (supporting information S2 E–G).

### Changes in the patterns of expression of some proneural genes

To examine at what stage the InR activates genes involved in PNS formation, tests were undertaken using a *tub-GAL80^ts^,UAS-InR* strain, and the receptor expression was induced at various times of development (L1, L2, L3) putting the strain at the permissive temperature every 8 hours. The results show that InR acts at an early stage, at the end of the second instar and early third instar just prior to SOP formation (data not shown).

At this stage, proliferation induced by the InR is very low and no significant difference in wing disc size is observed with and without induction of the receptor.

To visualize the effect of InR and FOXO on SOP formation and proneural gene expression *InR* was overexpressed in the *dpp* domain. Wing discs were stained with Ac antibodies to identify the proneural clusters, and stained with Sens antibodies to detect SOPs.

**Figure 4:**
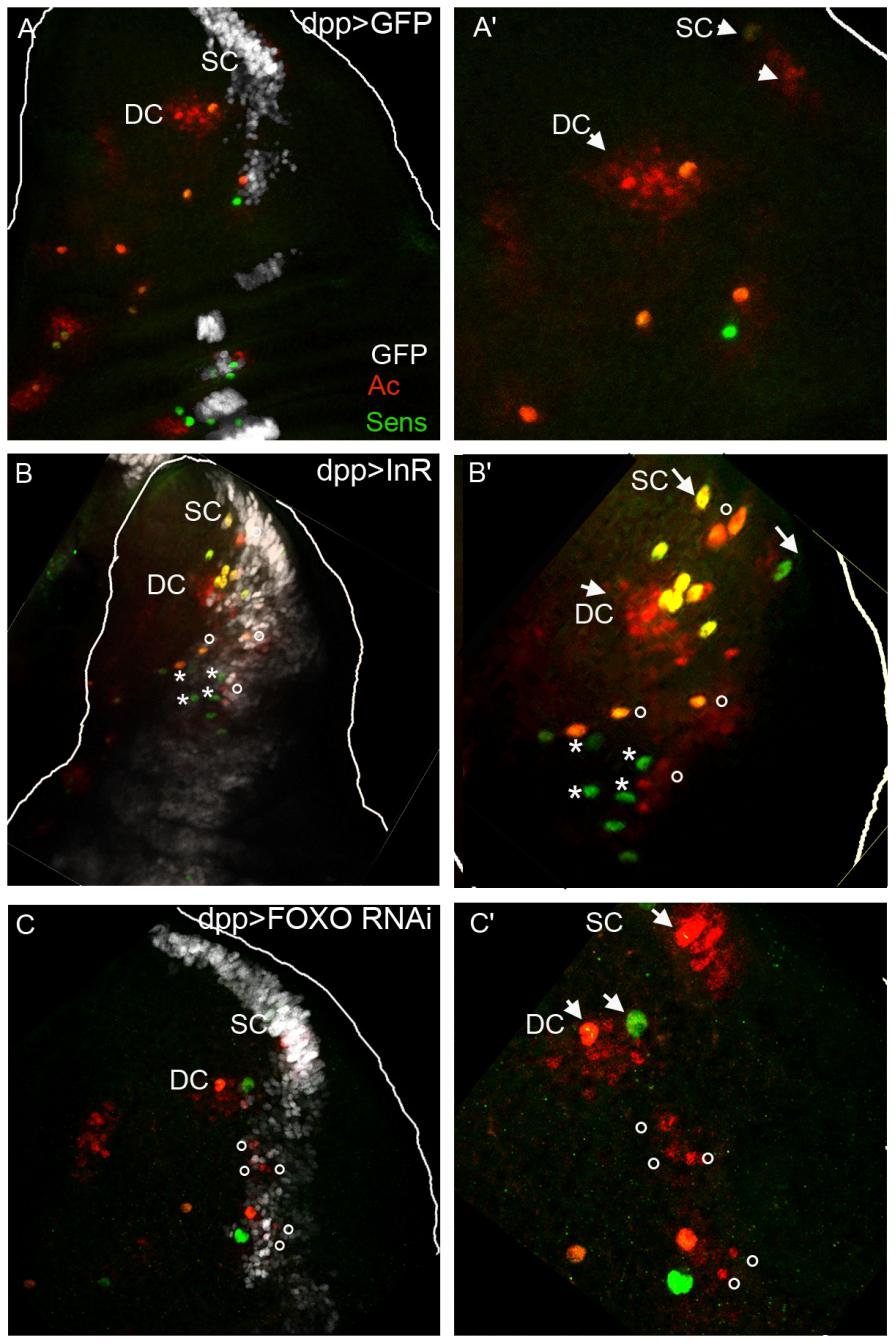
Induction by *InR* and *FOXO RNAi* of Ac and Sens. Confocal images of third instar wing discs stained with Ac (red) and Sens (green) antibodies. All disk are oriented A/P (antero/posterior) from left to right: **A**) *dpp>GFP* which serves as control, **B**) *dpp>GFP,InR* and **C**) *dpp>GFP,FOXO RNAi.* **A' B' C'** are enlarged views of DC and SC clusters respectively. Note the ectopic expression of Ac (circle) and Sens (asterisk) cells along the A/P boundary in **B** and **C** identified by *dpp>GFP*.

Acquired confocal images were used to visualize expression of Ac and Sens (Figures 4A–B'). A significant increase in Ac and Sens staining was observed supporting the possibility that InR can induce extra SOPs at the antero-posterior (A/P) boundary. In the adult, ectopic macrochaetes were observed on the scutellum (Figure 2F). However, it is difficult to establish if this is due to a change in cell fate due to ectopic Ac expression or if these extra macrochaetes arise in potential clusters in the area (Figures 4A–B).

We undertook a study of the appearance of SOP formation using Ac and Sens antibodies in the wild type and in larvae in which *InR* was overexpressed. No staining was observed at the end of the second larval instar. In early wild type and *InR* overexpressed larvae third instar (35–40h before puparium formation (BPF)) the cluster marked with Ac was clearly stained with higher Ac staining in the future SOP. No Sens staining was observed. No differences between the two strains were observed (supporting information S3). At later stages (15–20h BPF) Ac staining was still clearly observed in the test strain and one SOP marked by Sens in the pDC and pSC cluster. In the *InR* strain at the same stage Ac staining was still observed. The cluster did not seem to be larger than in the test. However 2 to 6 Sens staining cells were observed at the level of the pDC and pSC clusters. This was correlated with the number of additional macrochaetes observed in this strain.

To evaluate the function of FOXO, *FOXO RNAi* was overexpressed in *dpp* sequences. *FOXO RNAi* overexpression led to induction of Ac (Figures 4C–C'), suggesting that Ac induction is linked to the absence of nuclear FOXO.

**Figure 5:**
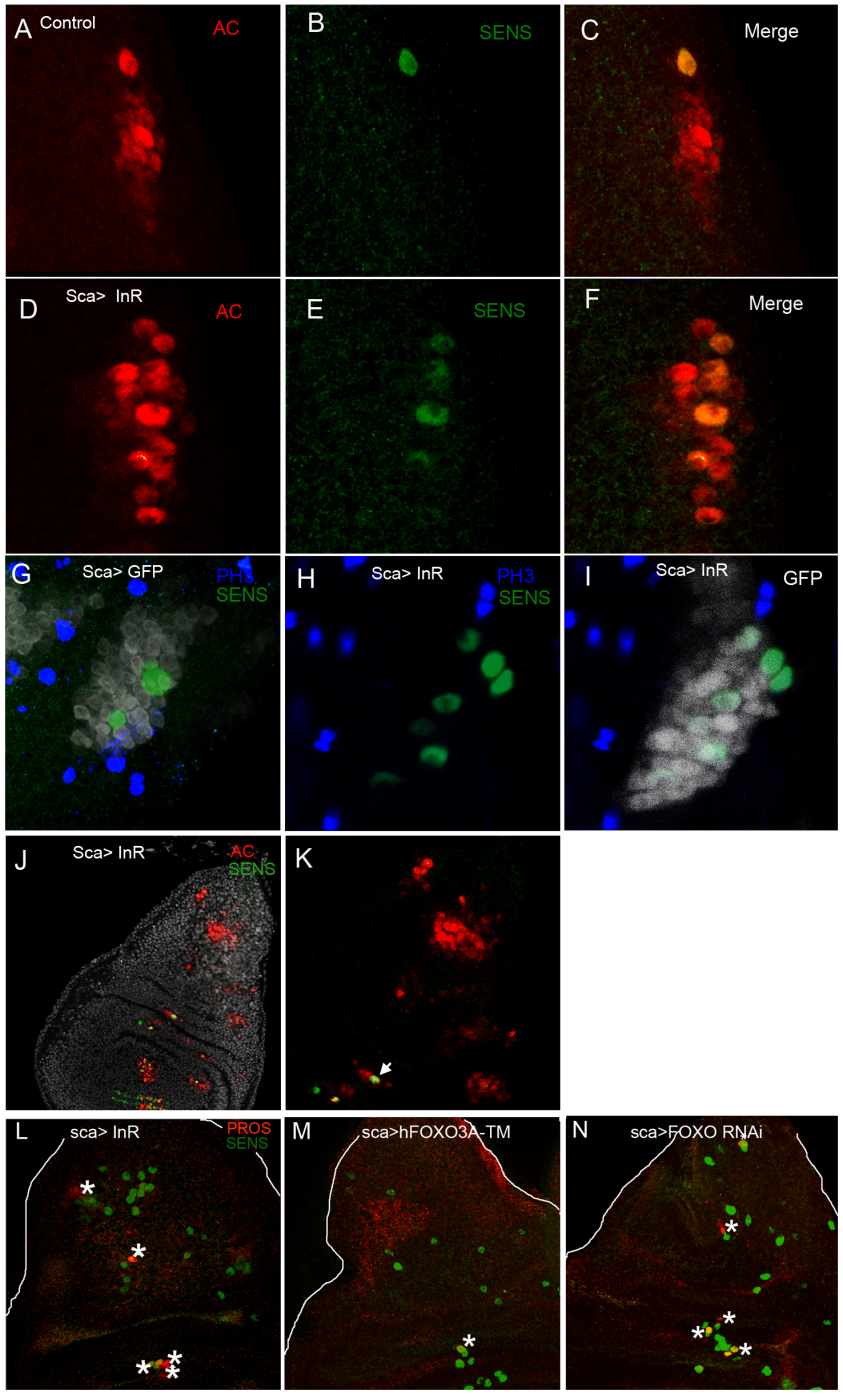
*InR* activates Ac and Sens expression and accelerates the time of sensory organ development. Confocal sections of the SC cluster expressing Ac (red) and Sens (green) in the control **(A–C)** and in *sca>InR* **(D–F).** Note that when *InR* is overexpressed the number of cells labeled is higher (**F**) and the signal is stronger (**D**). Proliferation was tested in **G, H** by labeling with anti-PH3 antibodies. In *tub-GAL80^ts^* and *sca>GFP*, 2 SOPs are labeled in the SC cluster (**G**). In *tub-GAL80^ts^, sca>InR,GFP* (**H, I**) 7 SOPs have emerged, but no mitotic activity is detected. Inside the cluster, cells are blocked in G2 as shown in [29]. In **J, K** (detailed notum) *sca>InR* early L3 wing disc shows strong Ac (red) labeling in most proneuronal clusters as usually observed in wild-type disks. No Sens (green) is detected in the DC or SC clusters when it is already visible in SOP at the DV boundary and hinge (arrow). Note that Sens does not appear earlier in SC and DC clusters on an L3 wing disc when *InR* was overexpressed. Wing imaginal disc from middle third instar larvae stained with anti-Sens and anti-Pro antibodies which specifically mark pIIb cells. In **L** *sca>InR* and **N** *sca>FOXO RNAi*, pIIb cells were detected (asterisk). On the contrary in **M** *sca>hFOXO3A-TM*, only one pIIb dividing cell is seen at the hinge. Activation of the InR pathway accelerates the time of development after SOPs were formed.

**Figure 6:**
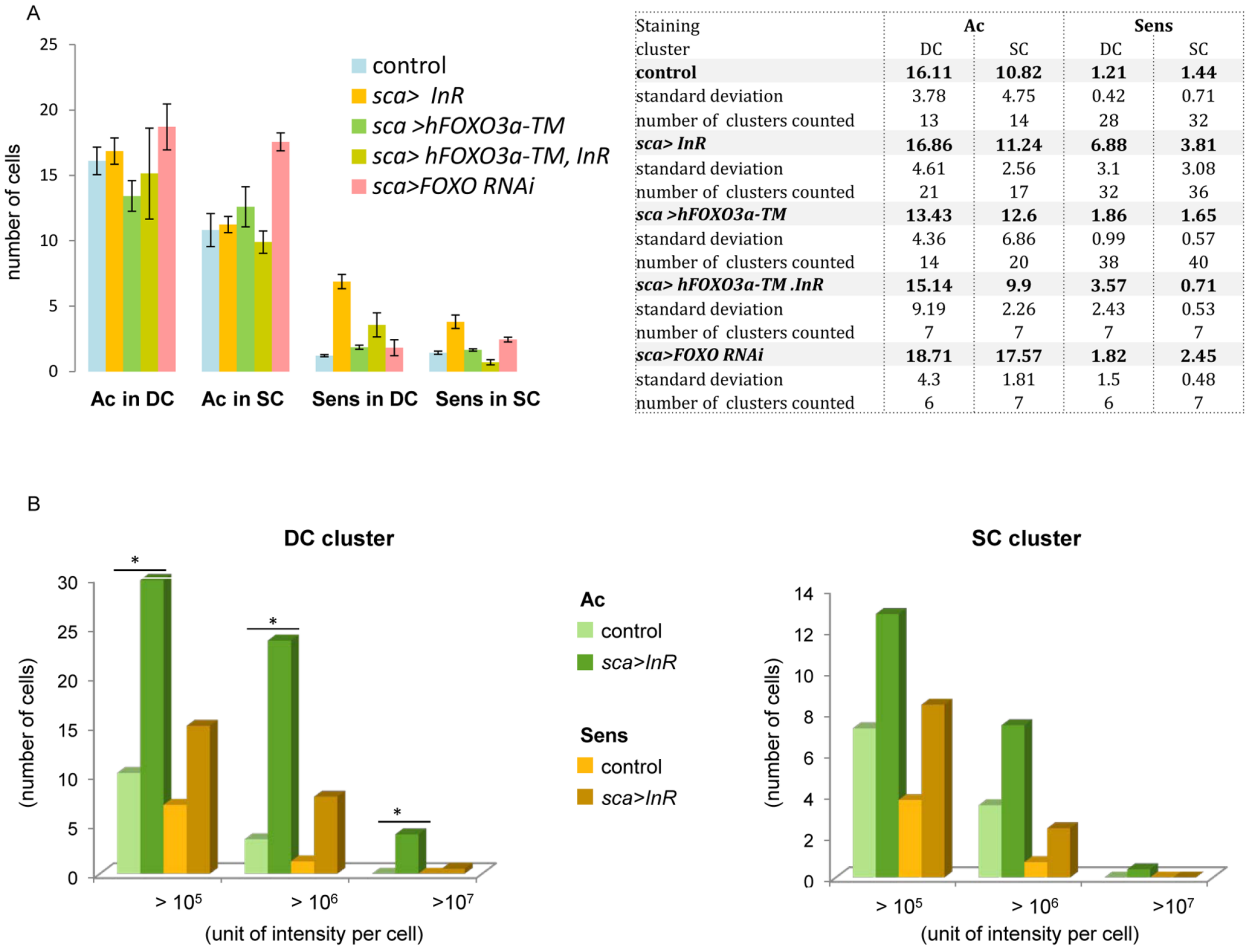
Relative quantification on Ac and Sens. **A.** Immunofluorescence labeling on wing imaginal discs performed with antibodies against Ac and Sens on *sca>GFP* (control), *sca>InR, sca>hFOXO^3A-TM^*, *sca>InR; hFOXO^3A-TM^* and *sca>FOXO RNAi*, showed higher staining of Sens when *InR* or *FOXO RNAi* were overexpressed. The Table presents the number of cells positive for Ac and Sens for the four genotypes for the DC and SC clusters. Data represent mean ± SEM. **B.** To evaluate the differences in the level of Ac and Sens expression in each cell in the DC and SC clusters between the genotypes, a relative quantification on confocal acquired images was performed after immunofluorescence labeling conducted simultaneously in control (*sca>GFP*), and *sca>InR* wing discs of the same age. The figure presents the number of cells in each cluster containing >10^5^; >10^6^; >10^7^ intensity units/cell. *p<0.05.

To better understand the effect of InR and FOXO on SOP formation we estimated the size of the proneuronal cluster in third instar larvae of the *sca>GFP* genotype (control), of *sca>InR*, of *sca>hFOXO^3a-TM^,* of *sca>Inr, hFOXO^3a-TM^* and of *sca>FOXO RNAi* genotypes and stained mid third instar wing discs with Ac and Sens antibodies. The number of Ac and Sens-cells corresponding to each genotype were quantified on confocal images (Figures 5A–F, 6A). When *InR* was overexpressed the number of cells labeled was higher than in the control and the signal was stronger. Cells positive for Ac and Sens showed different levels of expression, and the number of “high level Ac-expressing cells” was estimated for the genotypes tested (data not shown).

Since the number of Ac-positive cells defines the size of the proneuronal cluster, we propose that the increase in cluster size in *sca>InR* provides an estimation of the rate of proliferation of these cells, and of the decrease when *FOXO* is overexpressed. Were proliferation to only explain the increase of bristle number in *sca>InR*, the increase in the number of Ac and of Sens-positive cells should be comparable. This is not the case and the increase in Sens-positive cells is much higher than would be expected if only proliferation were to account for the phenomenon. The number of cells expressing Ac is fairly similar in the control strain and in the *UAS-InR,sca-GAL4* genotype, but the ratio of the number of Sens-expressing cells over Ac-expressing cells increased by 543% for DC and by 254% for SC when *InR* was overexpressed (Figure 6A). The size of the proneuronal cluster and the number of SOPs were examined using anti-Ac and anti-Sens antibodies when both *InR* and *FOXO* where overexpressed in *sca* sequences. Such flies displayed an intermediate phenotype (Figure 2H). Moreover, the number of “high level Ac-expressing cells” and the number of Sens cells were much lower than when only the receptor was overexpressed (Figure 6A), confirming the hypothesis that both genes act mainly at the same level but in opposite directions during development of PNS in SOP formation. However overexpression of *sca>hFOXO^3a-TM^* alone did not lead to a decrease in Sens-expressing cells, as expected by examining the adult phenotype.

We confirmed that Sens-positive cells are not due to an increase in cell division of SOPs when *InR* is overexpressed, as estimated by the rate of mitosis with anti-phospho-histone H3 (PH3) antibodies in mid third instar larvae (Figure 5G–I). No staining was observed, indicating that cells are blocked in G2 as reported [29]. No additional division occurred in the scutellar cluster of third instar larvae when *InR* was overexpressed. This confirmed the result that showed that the number of Ac expressing cells is comparable in both genotypes and that no proliferation occurred.

As already mentioned, high levels of Ac were observed in some cells of the proneuronal cluster when *InR* was overexpressed.

One hypothesis is that InR directly or indirectly causes induction of Ac in some cells of the proneuronal cluster. This increase amplified by self-stimulation of Ac and Sc, changes the fate of the cell that becomes an SOP instead of an epidermal cell.

To test this hypothesis the level of Ac and Sens in each cell of the proneural cluster was evaluated on several wing discs. Four to six discs of the *sca>GFP* genotype and the *sca>InR* genotype were used. Confocally-acquired images after immunofluorescence were analyzed with 3D visualization of reconstructed images with the Imaris software (Bitplane Scientific Software). Quantification of the number of voxels in each cell for the two fluorochromes (Cy3 and Cy5) corresponding to Ac and Sens expression was performed (see Materials and Methods). Once the background had been subtracted, 3 different levels of intensity were arbitrarily chosen from 10^5^ to 10^7^ intensity units per cell. Figure 6B, presents as a diagram, the results for DC and SC clusters confirming different levels of expression. It can be assumed that already at 10^5^ intensity unit per cell, Ac or Sens staining are physiologically significant. So far it had been shown that Sens is highly expressed in 2 SOPs in DC macrochaetes and in 2 SOPs in SC macrochaetes in the third larval instar [30]. However, our results indicate that some cells express significant amounts of Sens in the control genotype (*sca>GFP*). Looking at the numbers (supporting information S4), the ratio of the number of positive Sens-expressing cells in *sca>InR* over the number in the *sca>GFP* control cells reaches 2.14 for DC and 2.24 for SC clusters at 10^5^ intensity unit per cell. It then increases dramatically, as for the 10^6^ level indicating that a greater number of cells express high levels of Sens in the *InR* background. At 10^6^ a ratio of 6.26 for DC and 3.2 for the SC clusters was observed. At 10^7^, Sens staining was visible in the DC clusters in *sca>InR* but not in the control strain (Figure 6B).

The increase in level of Sens could be the consequence of an increase of Ac when *InR* is overexpressed (Figure 6B). Indeed the ratio of the level of Ac in *sca>InR* over the control is of 1.76 for the SC clusters and 2.91 for the DC clusters at 10^5^, and reaches 2.11 for the SC and 6.77 for the DC clusters at 10^6^. For Ac, like for Sens, Ac staining is observed at 10^7^ in SC clusters as well as in DC clusters. Thus, the number of cells expressing a high level of Ac is significantly higher in *sca>InR* and is compatible with the hypothesis that *InR* is involved in Ac up-regulation in the proneural cluster. This would explain the increase in Sens staining and the increase in SOP number.

**Figure 7:**
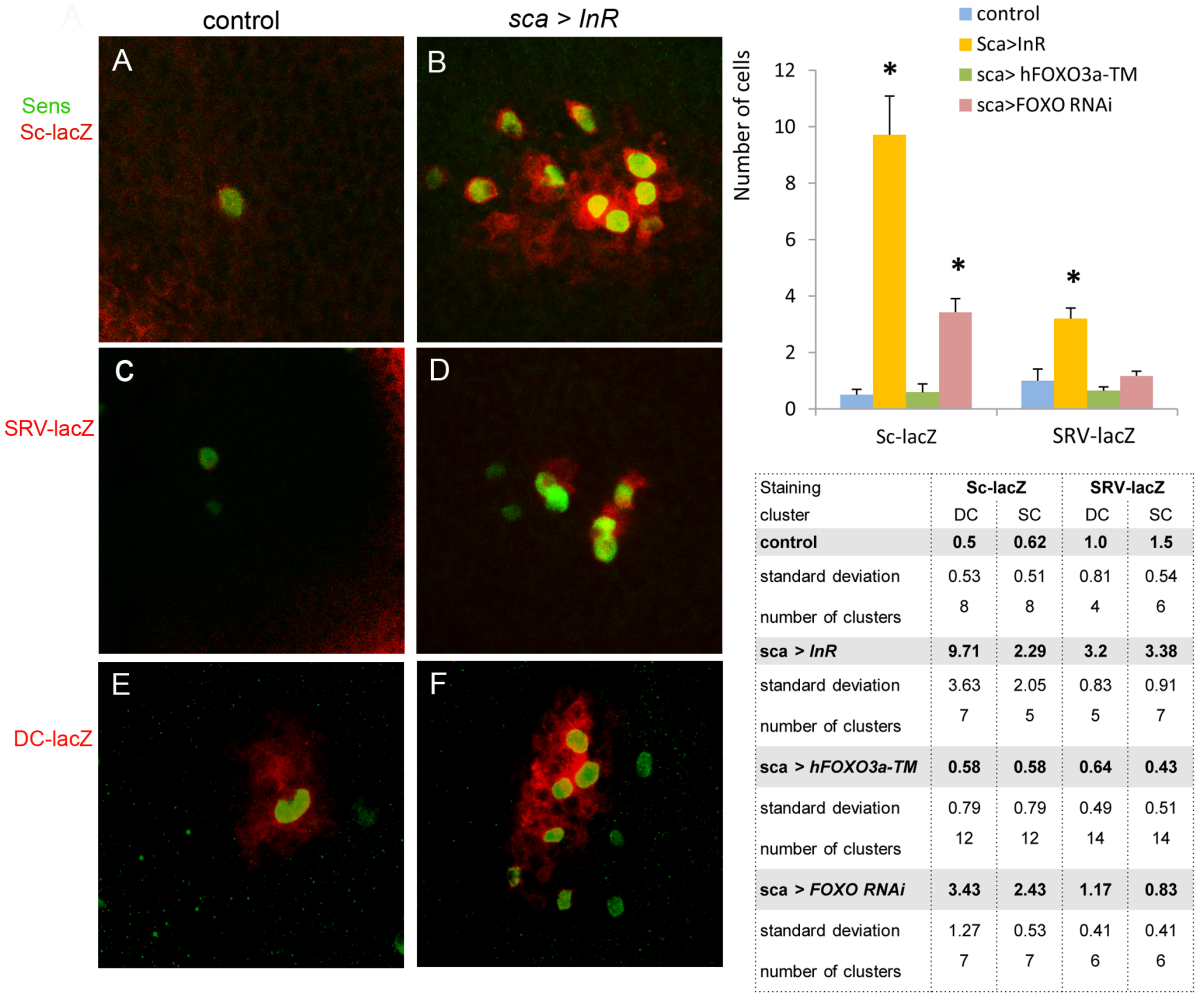
*sc* enhancers are activated by *InR.* On the left side the figure presents the DC cluster in control (*sca>GFP*), and when *InR* was overexpressed. Several additional Sens-positive cells are detected in the cluster (**B, D, F**) compared to the control (**A, C, E**). Note that the *sc-lacZ*, *SRV-lacZ* and *DC-lacZ* reporters are activated in all the supplementary cells (**B, D, F**). All the discs are oriented A/P from left to right. On the right side quantification of the cells expressing *sc-lacZ* and *SRV-lacZ* in the DC cluster. A comparison of the control genotypes, *sca>InR* and *sca>FOXO RNAi* confirms that *InR* overexpression and *FOXO* underexpression increase the activity of these enhancers. The average number of cells in the DC and SC clusters positive for the *sc-lacZ* and *SRV-lacZ* reporters, are presented in the Table. The results include the *sca>hFOXO^3a-TM^* genotype. Data represent mean ± SEM. *p<0.05.

To test the hypothesis that the InR regulates proneural gene expression, three *sc* reporter strains were used. Their expressions were compared in a genetic wild-type context and when the *InR* or *FOXO RNAi* were induced in *sca-GAL4* (Figure 7). The *sc-lacZ* strain made possible to observe the activity of a 3.8 kb sequence of the *sc* promoter, the *SRV-lacZ* an *sc* enhancer located in the promoter and expressed only in SOPs [31]. The *DC-lacZ* reporter is specific of DC macrochaetes.

Overexpression of *InR* induced in the three reporter strains an increase in positive lacZ spots (Figure 7).

The striking result is that a larger number of cells are *lacZ*-positive for the 3.8 kb *sc* promoter. The ratio in *sca>InR* is 19.4 times higher compared to the control strain for the DC cluster and 3.7 times higher for the SC cluster. This could not be due to proliferation as we have observed that the size of the cluster (stained by Ac) was identical for the two strains at this stage. In addition most of these 3.8 kb *sc-lacZ* were also Sens positive, and represent potential SOPs (Figure 7).

The results with the *SRV-lacZ* reporter showed that it is expressed 3.2 times more strongly in *sca>InR* than in the control strain for the DC cluster, and 2.2 for the SC cluster (Figure 7). This suggests that *InR* activates the *SRV* enhancer (Figures 7C–D). It also shows that *InR* induces other enhancers located inside the 3.8 kb *sc* promoter since the induction of *sc-lacZ* by *InR* is much higher than by *SRV* which marks only the SOPs. This is also the case for the DC enhancer (Figures 7E–F).

In *sca>hFOXO^3a-TM^* no significant differences with the control strain for *sc-lacZ* (Figure 7) was observed. For the *SRV-lacZ* strain a decrease in *lacZ* cells stained was observed, especially for the SC clusters. In addition in *sca>FOXO RNAi*, there was a significant increase in the number of *sc-lacZ* staining cells. Taken together these results are in favor of a regulation of *sc* by *InR* in proneural clusters.

### *InR* accelerates the time of sensory organ formation

It has been shown that increase or decrease of the components of the TOR/InR/FOXO pathway is closely linked to the temporal control of differentiation. Increase of InR signaling accelerates the process while a decrease delays the process at least during photoreceptor formation and chordotonal organ formation in the leg imaginal disc [17]. However, no effect of a *tsc1* mutant was observed by these authors in prehair formation in the pupal wing.

We tested whether *InR* could accelerate the time of SOP formation and division in the wing disc. In *sca>InR* wing discs, the first Sens-positive cells appear at the same time as in wild-type discs and not earlier (Figures 5J–K and supporting information S3). *InR* does not affect the appearance of SOPs. An effect of *InR* after the first division of SOP was also tested with anti-Prospero (anti-Pros) antibodies that marked pIIb cells. *UAS-InR,tub-GAL80^ts^* flies were used and *InR* was induced at the mid second instar. In wild-type wing discs, no Pros staining could be detected at the level of either DC or SC SOPs in late third instar larvae (data not shown). Staining appeared only in early pupae. On the contrary in *sca>InR* both DC and SC were marked, and in mid third instar larvae, Pros staining was already detected in DC SOP (Figure 5L). These results clearly indicate that overexpression of *InR* can accelerate the time of development of pIIb formation but this event cannot account for the excess number of positive Sens-stained cells observed for the same genotype. We tested whether *sca>hFOXO^3a-TM^* and *sca>FOXO RNAi* affect the time of development (Figures 5M–N). When *FOXO RNAi* was overexpressed, Pros staining appeared in third instar discs, as with the *InR* strain confirming the hypothesis that the absence of nuclear FOXO is involved in this process.

### Interaction between *InR/FOXO* and *sc*, *N*, and the *EGFR/Ras* pathway

If *InR* regulates *ac* and *sc* expression, genetic interaction between *InR*, *FOXO* and *sc* are expected. In parallel lateral inhibition is mediated by the N receptor [19], and it is presumed that this phenomenon is perturbed in an InR or FOXO background as it is in a *sc* overexpressed background.

To test a possible interaction between *InR* and *FOXO* with *sc*, two different *sc* mutants were used, *sc^10-1^*, which results in complete deletion of the *ac-sc* complex and *sc^1^*, which is a hypomorphic mutant due a transposable gypsy insertion.

Male *sc^10-1^* mutants are lethal at the adult stage but late pupae could be recovered and dissected. In *sca>InR*, male *sc^10-1^* mutants still displayed complete absence of microchaetes and macrochaetes confirming that the *InR* is upstream of *sc*, if necessary, is not sufficient for SOP formation (data not shown). Female heterozygotes for *sc^10-1^* presented a normal number of macrochaetes (Figure 3C). However in *sca>InR* females, *sc^10-1^* heterozygotes displayed a highly significant increase of all macrochaetes suggesting that *InR* could regulate the promoter of the wild-type allele (P<0.001) (Figure 3C and supporting information S1), as proposed in the preceding paragraphs. However there is a decrease in the number of macrochaetes (P<0.001) compared to *InR* overexpression in a *sc^+^* background confirming that a dosage effect exists with *sc.*

*sc^1^* males are viable and present normal DC macrochaetes but lack some aSC and almost all pSC macrochaetes (supporting information S1). In *sca>InR* the lack of SC macrochaetes in males was significantly diminished (p<0.001 for pSC), but in addition, the number of DC macrochaetes was significantly increased (Figure 3C and supporting information S1).

Yet another way to test genetic interaction is to overexpress both *InR* and *sc* in *sca*. A strong increase of the overexpressed *sc* phenotype was observed confirming interaction (Figures 2N–O). On the contrary, when *hFOXO^3a-TM^* and *sc* were both overexpressed, a decrease of the *sc* overexpressed phenotype was observed in some parts of the notum (Figure 2P). Taken together these results are in line with the hypothesis of an induction of *sc* by *InR* and repression by *FOXO.* These interactions could also be explained by the possibility that InR/FOXO and *sc* share a common target or cooperate to induce these targets.

The N receptor plays a major role in the choice of cell fate in the *Drosophila* epidermis, singling out the neural precursor in a group of equivalent cells. A cell mutated for *N* autonomously adopts a neural cell fate [32]. SOP will express higher amounts of Sc and the cells neighboring SOP will become epidermis because of the N pathway and of its effectors E(spl) that block Sc target expression [33], [34]. Positive genetic interaction between *InR* and *FOXO* should accentuate or decrease the neural phenotype.

If InR/FOXO acts on the expression of *ac* and *sc*, it is assumed that N function could be perturbed. A strong N mutant, *N^55e11^*, harboring an *N* deletion was used [19]. Mutant males are lethal; therefore interaction can be visualized only in females. *N^55e11^* heterozygote females present an increase in the number of aDC and aSC macrochaetes (Figure 3D and supporting information S1).

In *sca>InR* a very significant increase in the number of all four macrochaetes was observed (p<0.001) not only in the *N^55e11^/+* females but also compared to the overexpressed *InR* in an *N^+^* genetic background (p<0.001). Moreover in *sca>InR RNAi* (p<0.001) a significant increase of aSC macrochaetes was observed in the *N^55e11^/+* background. Surprisingly, the knock-down of InR in the *N* heterozygotes similarly caused enhanced bristle formation, indicating that manipulation of InR signaling in a N sensitized background can have counter intuitive effects and that the integration of N and InR signaling is likely complex. These interactions could indicate that *InR* can accentuate the neurogenic phenotype of the *N* mutant perturbing lateral inhibition. In parallel, underexpression of *FOXO* either in *sca>FOXO RNAi* or in a *FOXO^25^/+* heterozygote background, induced, as does *InR*, a very significant increase in the neurogenic phenotype at the level of the aSC macrochaetes (p<0.001; Figure 3D and supporting information S1). This is an additional indication that the InR neurogenic phenotype is due to the cytoplasmic retention of FOXO but also that the *UAS-RNAi* strain mimics lack of *FOXO*.

Moreover in *sca>hFOXO^3a-TM^* in an *N^55e11^/+* background, the neurogenic phenotype was also very significantly increased (p<0.001) compared to *sca>hFOXO^3a-TM^* in a wild type background. However, surprisingly, overexpression of *InR RNAi* increases the number of aSC macrochaetes.

These results strongly suggest that *InR* and *FOXO* interact genetically both with *sc* and *N*. Nevertheless, the possibility that the effect of the InR/FOXO pathway on N might indirectly be the consequence of the action of these genes on *sc* expression, can be postulated.

The EGFR and the Ras/Raf pathway are involved in PNS formation. *EGFR* mutants display a decrease of DC macrochaetes but sometimes macrochaete duplication is observed [35]. This is confirmed using a dominant negative form of *EGFR*, overexpressed in *sca* sequences [24]. It seems that the absence of the EGFR signal fails to trigger the self-stimulatory *sc* loop [24].

Genetic interactions of InR and Ras, that transduces the EGFR pathway, have been suggested in different developmental processes and particularly in photoreceptor differentiation. Data showed that there is a crosstalk between the InR/TOR signaling and the EGFR pathway [36], [37]. Therefore, we tested whether interactions exist between the two pathways in macrochaete development. When 3 different constructs of the *EGFR RNAi* transgene were overexpressed with *sca-GAL4,* few duplications of aSC and aDC macrochaetes were observed (Figure 3B and supporting information S1B). The same observation has been described in *EFGR* hypomorphic mutants [35]. Much stronger phenotypes were observed when *sca>EGFR* was overexpressed (Figure 3B). In this case the number of aDC and aSC macrochaetes was significantly increased (p<0.001). In *sca>EGFR*, *InR* a significant increase in the number of macrochaetes is observed compared with overexpression of a single transgene, indicating that the two pathways genetically interact in this process. In addition in *sca>EGFR*, *FOXO3a* a significant decrease is observed compared to *sca>EGFR* confirming the interaction between the two pathways. However a significant increase was also observed in the *sca>EGFR RNAi*, *InR* cross. To better understand this result we overexpressed the *EGFR RNAi* strain with *FOXO3a* then with *FOXO RNAi*. The results were not significantly different from those with the *EGFR RNAi* strain alone. This suggests that the interaction observed between the *EGFR RNAi* and InR could be FOXO independent.

In the wing disc, Ras acts downstream the EGF receptor and it is assumed that *Ras^V12^* transduces the EGFR pathway [38]. Ras is a molecular switch between an inactive and active GTP-bound form. It induces cell proliferation and accelerates G1/S transitions but does not accelerate the rate of cell division, and moreover it interacts with the dMyc oncogene [39]. Overexpression of *Ras^V12^* using the *C253-GAL4* driver that constitutes a subset of the *sca* promoter, indeed produces an excess of all macrochaetes. In *C253-GAL4*>*InR*, *Ras^V12^* a significant increase in the effect of only one transgene is observed (Figures 2K–M). Interestingly the overexpression of *Ras^V12^* using the *C253-GAL4* driver in an *InR^EX15^* heterozygote background significantly decreases the overexpression of *Ras^V12^* by itself, suggesting that InR is required for proper Ras signaling. However, overexpression of *InR* in a heteozygote *Ras1* background did not reduce the number of macrochaetes compared to overexpression of InR alone probably due to the haplosufficiency of the mutation (supplementary information S1B). Taken together these data confirm the results observed with EGFR and the interaction between the two pathways.

## Discussion

We proposed a model in which the *InR* receptor plays a role in the development of the peripheral nervous system mainly through FOXO cell localization independently of its role in proliferation and apoptosis. The role of the InR/FOXO pathway appears early in PNS development before SOP formation. The use of different mutants involved in growth indicates that the TOR pathway does not play a major role in the phenotypes observed. Our results using genetic and molecular methods strongly suggest that InR/FOXO controls the level of proneuronal genes such as *ac*, *sc* and *Sens* early in PNS development. This explains the interaction observed with *N^55e11^*.

Several arguments indicate that the phenotypes observed when *InR* is overexpressed are not due, at least for the most part, to proliferation, growth or lack of apoptosis. First using anti-PH3 staining that allows to visualize mitotic cells, no extra mitoses are observed in the cluster. Overexpression of genes such as *dE2F1*, or *dacapo* did not lead to a significant increase or decrease in the number of macrochaetes. In addition co-expression of these genes with *InR* indicates no interaction. Moreover, the effects of *InR* and *FOXO* when overexpressed on respectively the increase and the decrease in cell number, could be estimated by the number of Ac-positive cells in the DC and SC clusters. No significant differences were observed between the control and the overexpressed strain (either *InR* or *FOXO*) in the number of cells positive for Ac. If the possibility that proliferation is somehow involved in cluster size cannot be discarded, it does not account for the effects observed since the ratio of Sens-positive cells when *InR* is overexpressed over the control strain is much higher than the ratio of Ac-positive cells. A similar role for FOXO in apoptosis could also be discarded on the same basis. No clear interactions were observed between *FOXO* and genes involved in inhibition of apoptosis like *diap^1^*.

Along the same line it has been shown by Bateman [17] that the InR/TOR pathway plays a role in controlling the time of neural differentiation. This has been observed in photoreceptor formation but also in the chordotonal organs of the leg that develop on the same basis as thoracic bristles. The dynamic formation of the SOPs, particularly after a block of InR signaling was undertaken. No differences were observed before the end third larval instar in the test and in the overexpressed strain. Only an increase in the number of positive Sens stained cells are observed in the *sca>InR* strain.

Using Pros staining that marks pIIb cells, we show that staining appears in the late third instar larvae at the level of DC SOPs in *sca>InR*; this is not observed in the control strain. In addition in *sca>FOXO RNAi* wing discs it also leads to Pros staining. This indicates that the time of differentiation is advanced in the *InR* strain through the absence of nuclear FOXO. However we verified that in very early third instar larvae the first scutellar SOP appears at the same time in the control and in the overexpressed strains and that no differences were observed in mid third instar.

In addition our observations show that the increase in the number of macrochaetes in *sca>InR* is independent of the TOR pathway since none of the members induces a similar phenotype as does InR or interacts either with InR or FOXO in this process (supporting information S5 and S1E). However, some interactions were observed with raptor and Rheb that could be the consequence for the latter of its role in PIIa and PIIb formation regulating N [14].

Are InR and FOXO acting on the same target in SOP formation? Several arguments are in favor of this possibility. First underexpression experiments (*InR* clones, *InR RNAi* or *FOXO RNAi* overexpression and *FOXO* homozygotes and even heterozygotes,) induce exactly opposite phenotypes. This is also true for overexpression experiments with *InR* and *hFOXO^3a-TM^*. Moreover overexpression of both transgenes leads to an intermediate phenotype, very different from the control phenotype (Figure 2H). Finally, overexpression of *InR* in a heterozygote *FOXO* mutant background leads to an increase in the number of macrochaetes compared to *InR* alone (Figure 3A). *FOXO* null flies are fully viable and do not usually display any phenotype [8]. However an increase in the number of pDC and aSC macrochaetes is observed in some *FOXO* homozygotes and even heterozygotes that are nor observed in the control strain (supporting information S1 and S2). This could indicate that FOXO function is in part dispensable. Even if the *InR/FOXO* double heterozygote is completely normal, the double null mutant InR/FOXO shows either an excess or a lack of macrochaetes, that is in favor of the hypothesis that InR acts through FOXO (supporting information S1E). *FOXO* null clones do not display any phenotype comparable to *FOXO RNAi* overexpression. However overexpression of *InR* in a *FOXO* null clone leads to stronger phenotypes than overexpression of *InR* alone in a clone (supporting information S2). Yet, we cannot exclude that part of the InR overexpression phenotype is not due to the absence of FOXO or its cytoplasmic retention.

The absence of FOXO, using *FOXO RNAi*, or its retention in the cytoplasm by *InR* or *Akt* overexpression produces the same neurogenic phenotypes that are exactly the opposite when nuclear *hFOXO^3a-TM^* is overexpressed. In addition overexpression of both *hFOXO^3a-TM^* and *InR* leads to a decrease in the number of highly positive Ac and Sens expressing cells compared to overexpression of *InR* alone. Finally, overexpression of *FOXO RNAi* in *dpp* regulatory sequences, induces Ac expression. All these results should be explained by the same molecular process. One possibility would be that InR/FOXO regulates one or several neural genes involved in cluster formation and maintenance. Our results are in favor of the hypothesis that genes of the *Ac/Sc* complex could be regulated by InR. Either InR via nuclear FOXO represses the Ac/Sc pathway, or FOXO activates a repressor of the pathway.

Since it has been well established that InR induces cell proliferation [40], [41], and FOXO reduces cell number [8], [10], it remains possible that these functions could affect the size of the proneural clusters when the genes are overexpressed. However, when the number of the Ac-positive cells in the DC and SC clusters in the different genotypes was estimated, it was not significantly different.

Several relevant arguments exist suggesting that InR is necessary for SOP formation and regulation of neural gene expression. (i) The phenotype of the overexpression experiments either with *InR* or with *InR RNAi* suggests that InR perturbs the normal pattern of singling out a cell in the proneural cluster that will become an SOP. The fact that the sensitive period occurs in the late second/beginning third instar is in accordance with this hypothesis. The phenotype of the *InR* null clones comfort this hypothesis. (ii) When *InR* is overexpressed the level of Ac is significantly higher. This is confirmed by the IMARIS technique that estimated that in this genotype, the number of cells with the highest scores (10^6^ and 10^7^ units) is larger than in the control strain. These “highly Ac-positive cells” seem to also be Sens positive cells indicating a correlation between the two events. (iii) In *sca>InR* the level of Sens, measured by the IMARIS technique is higher than in the test raising the possibility that InR regulates several neural genes independently. However another possibility would be that this high Sens expression level would be indirectly due to the induction by InR of a Sens-positive regulator such as *sc*. (iv) Several *sc* enhancers are regulated by *InR*, the *sc* promoter, and the *SRV* and *DC* enhancers. As *sc* is auto-regulated through its different enhancers, it is difficult to evaluate if a specific enhancer is involved although the effect on the 3.8 kb *sc* promoter is the most striking (Table in Figure 7). For FOXO the absence of FOXO using the *FOXO RNAi* strain shows that Ac is induced. The double expression of *InR* and *hFOXO^3a-TM^* produces an intermediate phenotype and decreases the effects of *InR,* on Ac and Sens expression. The results using the *sc* enhancers when *hFOXO^3a-TM^* is overexpressed showed that only a decrease in the expression of the *SRV* enhancer is observed. However, the phenotypes observed in *sca>hFOXO^3a-TM^* agree with the hypothesis of repression of *ac* and *sc* by *hFOXO^3a-TM^*. As expected, overexpression of *FOXO RNAi* induces *sc-lacZ* enhancer. (v) Overexpression of both *InR* and *sc* leads to a significant increase in the effect of a single transgene. This indicates that both transgenes have a common target; one of them could be *sc* itself. An opposite effect is observed with *hFOXO^3a-TM^*. This favors the model whereby *InR* and *FOXO* act in opposite ways on the *sc* target in SOP formation. (vi) Highly significant genetic interactions are observed between *sc* and *InR*, and *sc* and *FOXO*. (vii) Another gene *charlatan* (*chn*) which is both upstream and downstream of *sc* [42], strongly interacts genetically with *InR* (data not shown).

Lateral inhibition is determined by the activity of the N receptor. When *N* is mutated, cell fate changes and extra macrochaete singling appear. Using the *N* deletion (*N^55e11^*) to test possible genetic interaction with *InR* and with *FOXO* in heterozygote females, interaction was observed with the *InR RNAi* strain. Moreover strong interaction is observed with *InR* overexpression. This indicates that *InR* impairs lateral inhibition and cooperates with *N* in this process. In parallel, as for *Inr* overexpression, the absence of nuclear FOXO either using *FOXO^25^* homozygotes (or even heterozygotes) or *FOXO RNAi* overexpression induces an increase in the neurogenic phenotype. With this latter strain, tufted microchaetes were observed, indicating that FOXO could also act later in development (Figure 2J). Overexpression of *hFOXO^3a-TM^* displays highly significant interaction with *N^55e11^* as the neurogenic phenotype is increased compared to overexpression in a wild type background. However, overexpression of InR RNAi in a *N^55e11^* hterozygote background leads to a significant increase but only at the level of aSC, raising the possibility of a local interaction or appearing at a specific time for the different clusters.

Moreover the fact that there is no differences when Suppressor of Hairless (Su(H)) which transduces the N pathway, is expressed with or without the *InR*, indicates that lateral inhibition is not affected. In addition in the *InR* strain, Sens stained cells were clearly individualized and separated from one another. These results clearly indicate that InR and FOXO act with N on the choice of the cell that will become an SOP.

Strong genetic interaction exists between the InR and the EGFR/Ras pathways. EGFR has also been implicated in macrochaete development [35]. Indeed *EGFR* mutants and *EGFR* null clones display macrochaete phenotypes [35], [43]. This could be explained since in *EGFR* hypomorphic mutants the level of Sc is reduced in some clusters and increased in others suggesting a different requirement of EGFR for the different SOPs [24]. If *Ras^V12^* was overexpressed with an ubiquitous driver, *sc* was ectopically expressed [24]. Thus, Ac/Sc induction by Ras overrules lateral inhibition due to N. Moreover N downregulation enhances EGFR signaling [24]. These authors established a model of antagonist interaction between EGFR and N in which Ac/Sc activates both pathways that in turn act on the same SOP specific enhancers.

Moreover, the InR/TOR pathway regulates the expression of some of the components of the EGFR signaling pathway such as *argos*, *rhomboid* and *pointed* [37]. Our results suggest that both the InR and the EGFR/Ras pathways induce *sc* in a synergic manner and this further overrules the lateral inhibition mechanism induced by N. The fact that overexpression of *Ras^V12^* in an *InR* null heterozygote background significantly lowers the phenotype observed with *Ras^V12^* only, is in agreement with this hypothesis. The interactions observed with the *EGFR RNAi* strain seem to be FOXO independent (see supplementary information S2).

Taken together our results show that InR and several components of the pathway such as PTEN, Akt and FOXO are involved in PNS development independently of their role in growth, proliferation and delay in the time of neural differentiation. The function of InR in PNS development seems to be independent of TOR/4E-BP. FOXO cytoplasmic retention either by InR activation or by the use of *FOXO RNAi* produces opposite phenotypes suggesting that nuclear FOXO could be a repressor of PNS development. Our results using antibody staining and reporters of *sc* enhancers indicate that InR targets are the neural genes *ac*, *sc* and *sens*. However, as most of these neural genes display a complex co-regulation, it is difficult to demonstrate whether or not *sc* is the primary target of the pathway. A strong interaction is observed between the EGFR/Ras pathways and InR suggesting that both could act together to induce neural gene expression and this would explain the strong interaction observed between InR/FOXO and N.

## Materials and Methods

### Fly stains

Fruit flies were raised on a standard *Drosophila* medium at 21 or 25°C. Overexpression of genes in proneural clusters was carried out using the UAS/GAL4 system [44], with *sca*-*GAL4* [45] or *C253*-*GAL4* [46] drivers; earlier developmental drivers, *dpp-GAL4* (Bloomington Stock Center) and *pnr^MD237^-GAL4* (a gift of F. Schweisguth [47]) were also used. The *UAS*-*InR^WT^* [26], *UAS*-*InR^exel^* (exelexis), *UAS*-*InR^DN^* [48], *UAS*-*PTEN RNAi*, *UAS*-*EGFR RNAi, UAS-raptor RNAi*, *UAS-Rheb RNAi*, *UAS-diap1* and *UAS*-*GFP* (used as driver control) lines as well as the *N^55e11^* and s*c^1^* or s*c^10-1^* mutants were from the Bloomington Stock Center. Other RNAi lines, *UAS*-*InR RNAi*, *UAS*-*PTEN RNAi*, *UAS*-*FOXO RNAi* and *UAS*-*4E-BP RNAi,* were from the Vienna Drosophila RNAi Center (VDRC) Stock Center. The *UAS*-*dFOXO A* and *UAS*-*dFOXO B* were already described [10], as well as the *UAS*-*hFOXO^3a^* ^-*TM*^ [8], *UAS*-*dilp2* [48], *UAS*-*dAkt1* [49], *dFOXO^21^* and *dFOXO^25^* mutants [8], the *UAS*-*Tsc1*, *UAS-Tsc2* [50], *UAS*-*S6K* [51], *UAS*-*E2F-DP*,*UAS*-*P35* [52], *SRV-lacZ* [22] and *DC-lacZ* (a gift of P. Simpson). The *UAS*-*EGFR* [53], *UAS*-*Ras^V12^* [24] and the *3.8kb sc-lacZ* [46] lines were gifts of J. Modolell. Other fly lines were from the Bloomington Stock Center.

To determine the stage during which *InR* activates genes involved in PNS formation, *InR* overexpression was performed using a strain bearing *tub*-*GAL80^ts^* (Bloomington Stock Center), the *tub*-*GAL80^ts^,UAS-InR* line, that we crossed with the *sca-GAL4* driver strain. Fly crosses, embryonic and larval development were carried at 21°C, and larvae at different stages were transferred to 29°C to allow the expression of GAL4. Somatic clones were obtained using the FLP/FRT recombination system [25]. The *FRT82B-InR^ex15^* line [54] was crossed to the *y*,*w*; *Ubx-FLP; FRT82B-ubi-nls::GFP* (gift of M. Gho [55]) to generate *InR*-null somatic clones.

### Statistics in Figure 3

As indicated in the Table legend, the number of macrochaetes (n) on fly hemi-thoraces was counted at each position on the notum. The results were divided into 6 classes, and the average for each position is presented. As some values were small and some null, we chose the Fisher Exact Test to calculate p-values, with the “R” programming language. The letters indicate the strains that are compared. Different colors are used: blue letters for 0.05<p<0.01, red letters for 0.01<p<0.001, and black letters for p<0.001.

### Immuno-histochemistry and microscopy

Imaginal disc dissections were performed on wandering 3rd instar larvae in phosphate-buffered saline (PBS). Discs were kept on ice until fixation in 4% paraformaldehyde in PBS Primary antibodies were used at the following dilutions, monoclonal anti-Ac (mouse) 1:5, (DSHB); polyclonal anti-Sens (guinea pig) 1:3000, gift of H. Bellen; polyclonal anti-PH3 (rabbit) 1:500, (Upstate Biotech); monoclonal anti-β-Gal (mouse) 1:200 (DSHB). Fluorescence FITC, Cy3 and Cy5 (1:200) conjugated anti-rabbit, anti-guinea pig or anti-mouse secondary antibodies were purchased from Jackson Immunoresearch Laboratories. Stained specimens were mounted in Citifluor AF1 solution (CITIFLUOR Ltd) antifade Reagent and images were obtained with an LSM 710 confocal microscope. Images were processed with ImageJ and Photoshop.

### Level of Ac and Sens in each cell of the clusters

To score the differences in the level of Ac and Sens expression between the genotypes, a relative quantification on confocal-acquired images was performed after immunofluorescence labeling conducted simultaneously. Images were made on an LSM 710 confocal microscope with a 40x objective and a Z step of 0.5 µm for a correct sampling. Three dimensional representations of confocal image stacks were generated using the software package, Imaris (Bitplane Scientific Software, St. Paul, MN). After the confocal image stack was loaded into Imaris, intensity threshold for each channel was adjusted. The 3D images generated made it possible to identify cells and quantify the number of voxels in each cell for two fluorochromes (Cy3 and Cy5) corresponding to Ac and Sens expression. Quantity generated of signal measured in cells located next to the cluster was considered as background, and was eliminated from the intensity of the signal scored in each cell in the SC or DC clusters.

## Supporting Information

Supporting Information S1 **Details of** **Figure 3** **.** The number of the four kinds of macrochaetes (expressed as percentage) are detailed. The numbers go from zero to five and more. Figure 3 represents the average of the numbers for the four kinds of macrochaetes. The following code was used to compare the strains: one sign for 0.05<p<0.01, two signs for 0.01<p<0.001 and three signs for p<0.001.
(XLS)

Supporting Information S2 **(A)** An *InR* overexpressed clone in a *FOXO* null background. Tufted chaetes were observed that are never detected in a *FOXO* wild-type background. **(B)** The same genotype induces a much stronger phenotype on the thorax that only *InR* overexpression. **(C)** *sca>EGFR* thorax at 25°C. Some supernumerary macrochaetes were observed. In **(D)** both *EGFR* and *InR* were co-overexpressed. A significant increase in macrochaetes is observed compared to a single transgene either *EGFR* or *InR*. **(E)** *sca>E2F-DP,GFP* flies. No effect is observed on the thorax. Additional very thin macrochaetes are detected on the scutellum due to proliferation. **(F)** *sca>InR,Dap* thorax. The cross is lethal; the picture represents pupae. The number of supernumerary macrochaetes is comparable to overexpression of *InR* alone. **(G)** *sca>InR, DIAP* genotype. No effect is observed. **(H)** *FOXO^21^/FOXO^25^* flies at 25°C. A supernumerary macrochaete is detected. A similar phenotype could also be observed in heterozygotes (see supporting information S1).
(TIF)

Supporting Information S3 SC and DC clusters (mid third instar wing disc, 35 h BPF) stained with Sens **(A, B)** or with Sens and Ac **(A', B')** of the *sca>GFP* (control) **(A, A')** or *sca>InR* genotype **(B, B')**. No major differences between A and B. Overexpression of *InR* does not cause earlier Sens expression. At a later larval stage (20 h BPF) **(C–D'), t**he number of SENS expressing cells in SC and DC clusters is much higher in *sca>InR* genotype **(D,D')** than in *sca>GFP* **(C,C')**.
(TIF)

Supporting Information S4 **Quantification of the level of Ac and Sens per cell in DC and SC clusters by the IMARIS technique.** In Figure 6B the differences in the level of expression of Ac and Sens in each cell in the DC and SC clusters between the control (*sca>GFP*) and the *sca>InR* genotype were evaluated. The Figure presents the number of cells in each cluster containing >10^5^; >10^6^; >10^7^ intensity units/cell.
(TIF)

Supporting Information S5 **Genes involved in growth are not required for macrochaete development.** As in Figure 3 the number of macrochaetes for each position was determined and 6 classes were established. Experiments were performed at 25°C. The results are expressed in the medium percentage of the number of macrochaetes for a given position. The Fisher Exact Test was used. Underexpression and overexpression experiments were used to assess the role of genes of the TOR pathway on macrochaete development.
(TIF)
