# Supplementary figures and images for: The Insulin Receptor Is Required for the Development of the Drosophila Peripheral Nervous System
Source: PLoS One. 2013 Sep 12;8(9):e71857. doi: 10.1371/journal.pone.0071857 (PMC3772016; doi:10.1371/journal.pone.0071857)

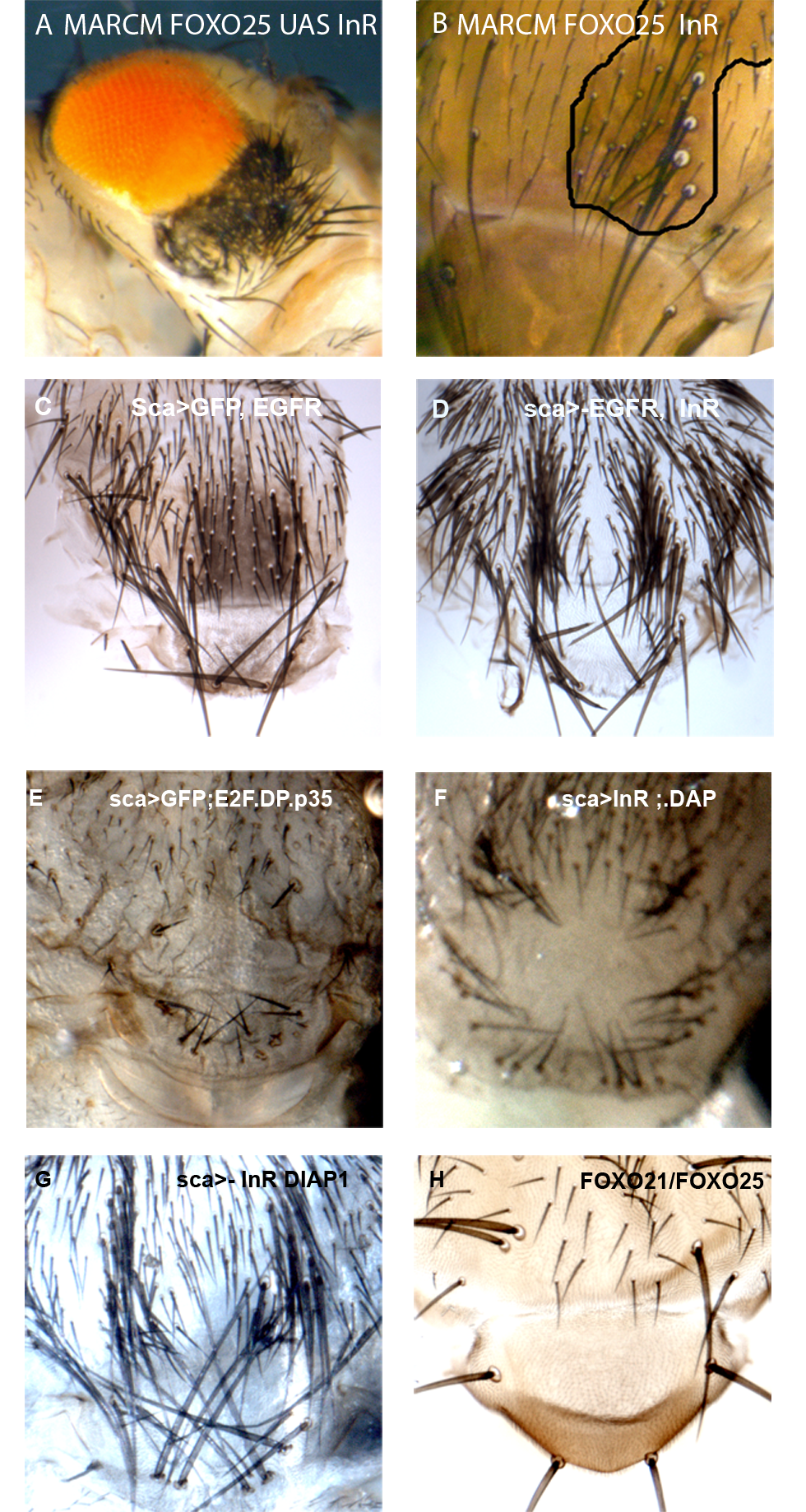

Supplement: Supporting Information S2 — (A) An InR overexpressed clone in a FOXO null background. Tufted chaetes were observed that are never detected in a FOXO wild-type background. (B) The same genotype induces a much stronger phenotype on the thorax that only InR overexpression. (C) sca>EGFR thorax at 25°C. Some supernumerary macrochaetes were observed. In (D) both EGFR and InR were co-overexpressed. A significant increase in macrochaetes is observed compared to a single transgene either EGFR or InR. (E) sca>E2F-DP,GFP flies. No effect is observed on the thorax. Additional very thin macrochaetes are detected on the scutellum due to proliferation. (F) sca>InR,Dap thorax. The cross is lethal; the picture represents pupae. The number of supernumerary macrochaetes is comparable to overexpression of InR alone. (G) sca>InR, DIAP genotype. No effect is observed. (H) FOXO21/FOXO25 flies at 25°C. A supernumerary macrochaete is detected. A similar phenotype could also be observed in heterozygotes (see supporting information S1). (TIF) [file pone.0071857.s002.tif]

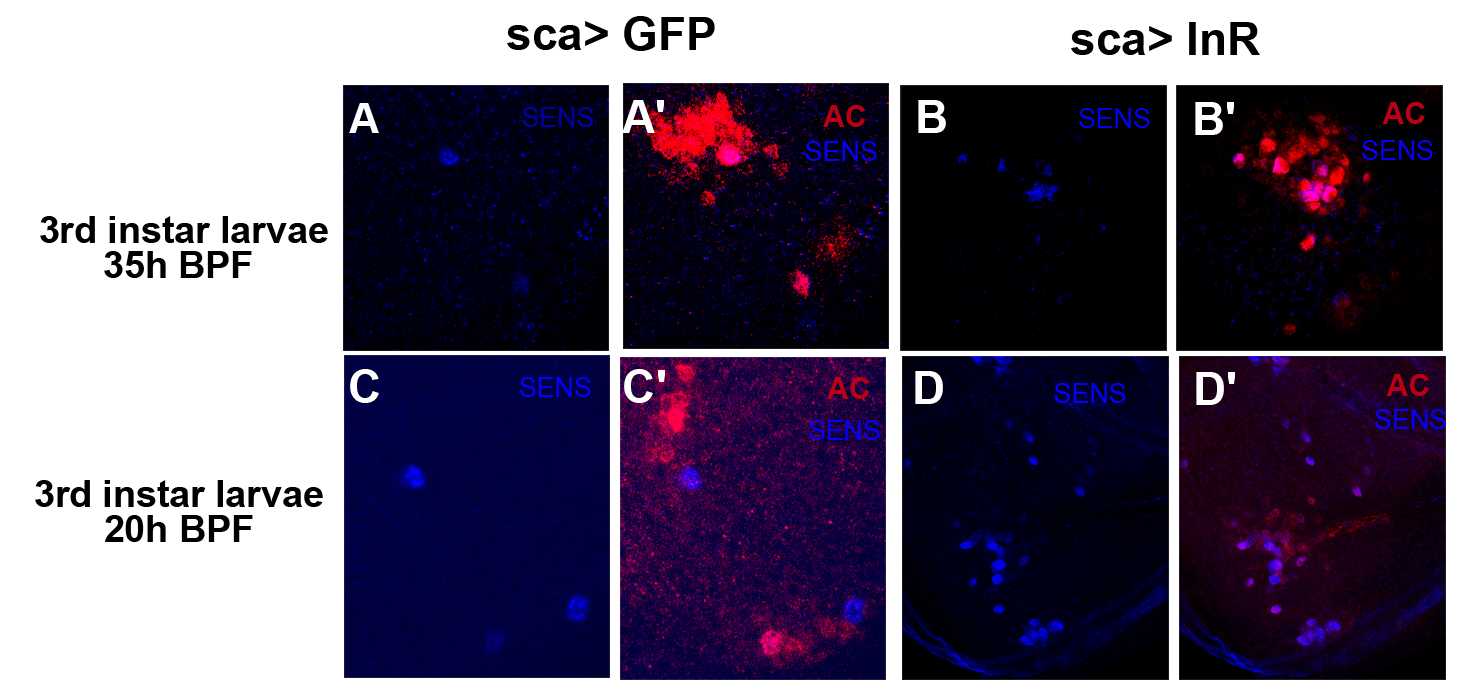

Supplement: Supporting Information S3 — SC and DC clusters (mid third instar wing disc, 35 h BPF) stained with Sens (A, B) or with Sens and Ac (A', B') of the sca>GFP (control) (A, A') or sca>InR genotype (B, B'). No major differences between A and B. Overexpression of InR does not cause earlier Sens expression. At a later larval stage (20 h BPF) (C–D'), the number of SENS expressing cells in SC and DC clusters is much higher in sca>InR genotype (D,D') than in sca>GFP (C,C'). (TIF) [file pone.0071857.s003.tif]

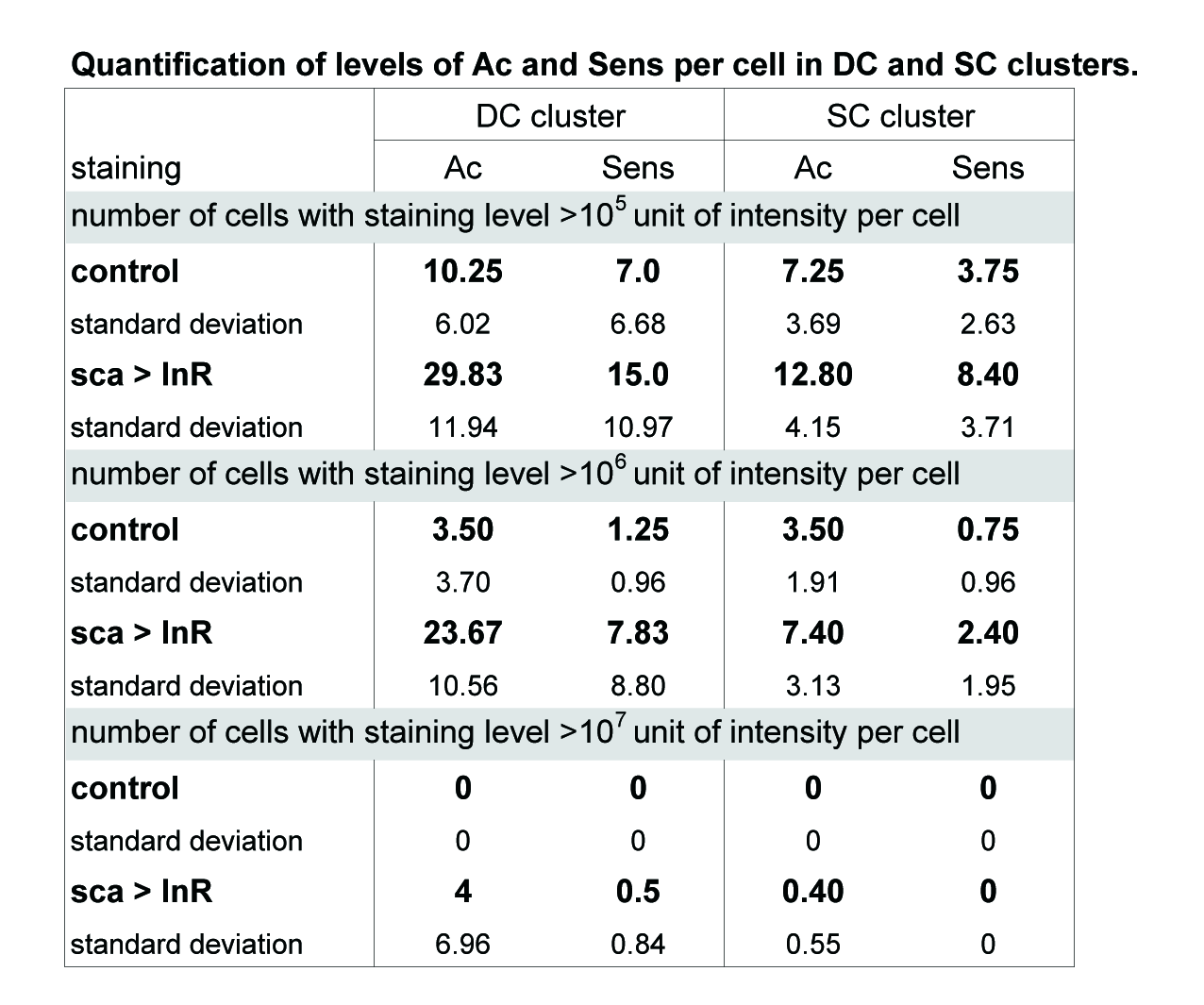

Supplement: Supporting Information S4 — Quantification of the level of Ac and Sens per cell in DC and SC clusters by the IMARIS technique. In Figure 6B the differences in the level of expression of Ac and Sens in each cell in the DC and SC clusters between the control (sca>GFP) and the sca>InR genotype were evaluated. The Figure presents the number of cells in each cluster containing >105; >106; >107 intensity units/cell. (TIF) [file pone.0071857.s004.tif]

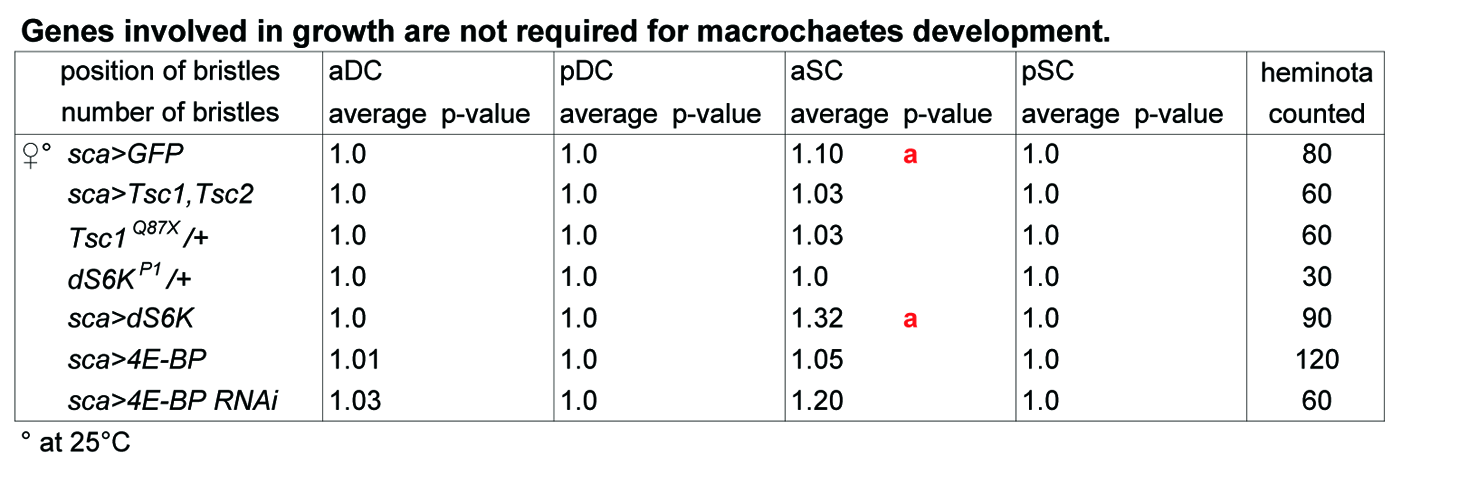

Supplement: Supporting Information S5 — Genes involved in growth are not required for macrochaete development. As in Figure 3 the number of macrochaetes for each position was determined and 6 classes were established. Experiments were performed at 25°C. The results are expressed in the medium percentage of the number of macrochaetes for a given position. The Fisher Exact Test was used. Underexpression and overexpression experiments were used to assess the role of genes of the TOR pathway on macrochaete development. (TIF) [file pone.0071857.s005.tif]
